# Transcriptional and immunohistological assessment of immune infiltration in pancreatic cancer

**DOI:** 10.1371/journal.pone.0238380

**Published:** 2020-08-31

**Authors:** Brady Bernard, Venkatesh Rajamanickam, Christopher Dubay, Brian Piening, Emilio Alonso, Zeljka Jutric, Ephraim Tang, Pippa Newell, Paul Hansen, Terry Medler, Andrew Gunderson, Kristina Young, Carlo Bifulco, Joanna Pucliowska, Marka R. Crittenden, Michael J. Gough

**Affiliations:** 1 Earle A. Chiles Research Institute, Providence Cancer Institute, Providence Portland Medical Center, Portland, Oregon, United States of America; 2 Liver and Pancreatic Surgery, Providence Cancer Institute, Portland, Oregon, United States of America; 3 The Oregon Clinic, Portland, Oregon, United States of America; 4 Pathology, Providence Portland Medical Center, Portland, Oregon, United States of America; Centro Nacional de Investigaciones Oncologicas, SPAIN

## Abstract

Pancreatic adenocarcinoma is characterized by a complex tumor environment with a wide diversity of infiltrating stromal and immune cell types that impact the tumor response to conventional treatments. However, even in this poorly responsive tumor the extent of T cell infiltration as determined by quantitative immunohistology is a candidate prognostic factor for patient outcome. As such, even more comprehensive immunophenotyping of the tumor environment, such as immune cell type deconvolution via inference models based on gene expression profiling, holds significant promise. We hypothesized that RNA-Seq can provide a comprehensive alternative to quantitative immunohistology for immunophenotyping pancreatic cancer. We performed RNA-Seq on a prospective cohort of pancreatic tumor specimens and compared multiple approaches for gene expression-based immunophenotyping analysis compared to quantitative immunohistology. Our analyses demonstrated that while gene expression analyses provide additional information on the complexity of the tumor immune environment, they are limited in sensitivity by the low overall immune infiltrate in pancreatic cancer. As an alternative approach, we identified a set of genes that were enriched in highly T cell infiltrated pancreatic tumors, and demonstrate that these can identify patients with improved outcome in a reference population. These data demonstrate that the poor immune infiltrate in pancreatic cancer can present problems for analyses that use gene expression-based tools; however, there remains enormous potential in using these approaches to understand the relationships between diverse patterns of infiltrating cells and their impact on patient treatment outcomes.

## Introduction

Pancreatic cancer is commonly characterized by extensive desmoplastic stroma and an environment that is poorly supportive of adaptive immune responses, yet like many other cancers, the degree of T cell infiltrate in pancreatic tumors is correlated with patient outcome [1–4]. T cells in pancreatic tumors face an array of suppressive mechanisms that can limit their ability to control tumors, and it would be beneficial to understand the relationship between T cell infiltration and the presence of other immune populations that positively or negatively regulate immune responses. For this reason, there is significant effort in the field to understand and manipulate the complex immune environment of tumors.

Quantitative immunohistochemistry (IHC) has long represented the gold standard by which tumor infiltrating immune populations can be assessed, and recent advances in multispectral IHC combined with automated image analysis have made possible an unprecedented ability to map out the immune environment of tumors. However, these approaches are limited by the availability and quality of antibodies, and complex multispectral panels require extensive validation to confirm the specificity and selectivity of binding. Recently, multiple groups have shown that the quantity of a diverse array of infiltrating immune cell types in a specimen can be inferred based on characteristic gene expression patterns unique to or enriched in specific cell types [5, 6]. In theory, a single RNA sequencing (RNA-Seq) analysis of preserved tissue can provide an assessment of immune cell infiltration as well as other information such as the cytokine and chemokine balance that may be regulating cell entry and retention in the tissue, together with candidate features of the cancer cells that orchestrate this environment. The addition of simultaneous whole exome sequencing can permit comprehensive profiling of cancer driver mutations, immune targetable mutations, as well as a personalized understanding of the patient’s immune profile [7, 8]. However, it remains unclear whether IHC and gene expression-based immune assessment approaches are highly concordant. For example, the utility of RNA-Seq in tumor profiling can be limited by a range of unique factors including degradation of transcripts in excised human tissues and by common tumor preservatives (e.g. formalin) and the ability to detect low-abundant transcripts. The latter is of particular concern in pancreatic cancer, which can have a relatively low infiltration of critical cell types such as CD8 T cells.

Thus far we are not aware of any studies that have directly compared IHC quantification of immune infiltration to RNA-Seq-based analyses in pancreatic cancer. In this study we aim to directly compare conventional IHC and gene expression-based approaches to characterize the immune environment of pancreatic cancer. We hypothesize that RNA-Seq analysis can provide a comprehensive alternative to quantitative IHC for immunophenotyping pancreatic cancer. We performed RNA-Seq on a prospective cohort of 39 pancreatic adenocarcinoma patient tumors with matched quantitative IHC, and evaluated approaches to quantify infiltrating immune cells using gene expression data. We found limited agreement between IHC and RNA-Seq analysis of infiltrating cells, however concordance was greatest when multiple cell types were aggregated to identify a mixed population, such as CD3^+^ T cells from a combination of CD4^+^, CD8^+^, and other cell types that express CD3. This aggregation may overcome the limitation of low T cell-derived RNA transcripts in poorly-immune infiltrated tumors. As an alternative, we identified gene signatures that were enriched in highly T cell infiltrated tumors that are associated with increased disease-free survival in other patient cohorts. These data demonstrate that immune infiltration remains an important predictor of outcome in pancreatic cancer patients, and that RNA analysis can provide an important addition to IHC data to understand the complexities of the immune environment that influence patient outcome.

## Methods

### Quantitative immunohistology for infiltrating immune cells in pancreatic cancer

We conducted a prospective cohort study of resectable pancreatic masses to determine which immunologic parameters have prognostic value. All procedures were approved under Providence Portland Medical Center Institutional Review Board, with approval number IRB 10–037, and patients provided written informed consent. We restricted our analysis to adult patients who underwent surgical resection for pancreatic masses. Patients were recruited from June 2010 to November 2014 at Providence Portland Medical Center in Portland, OR, where the research was conducted. Inclusion criteria included patients 18 years or older who had a diagnosis of a pancreatic or ampullary mass who were scheduled for surgical resection. Patients had to be able to give informed consent and could not have a diagnosis of a prior malignancy unless they were disease free for 10 years. We included patients who were subsequently determined to have other histologies. Demographics and survival of these patients are outlined in Table 1. Prior studies on this cohort had identified a positive correlation between CD3+ T cell infiltrate and overall survival by Multivariate Cox modeling and univariate analysis [1], so this sample set was applied for additional genomic analysis. Additional power calculations were not performed. Tumor infiltrating immune cells were quantified by immunohistochemistry and quantitative digital image analysis for CD3^+^, CD68^+^, and CD8^+^ cells as previously described [1]. Infiltrating cells were quantified from whole slide digital images scanned at 20x resolution (Leica SCN400). Regions of interest were defined with Pathologist guidance using Definiens Tissue Studio (Definiens Inc), and the automated algorithm used the immunohistology staining combined with nuclear counterstain to count total cells and positive cells to report a marker positive cell density per mm^2^ tissue for each patient. Primary outcome was overall survival.

**Table 1:** Demographics of patients on the study.

*Characteristic*	*Data*
***Pancreatic Adenocarcinoma*, *n***	**75**
*Age*, *median (range)*, *y*	66 (25–87)
*Male*, *n (%)*	43 (57%)
*Female*, *n (%)*	32 (43%)
***Neoadjuvant treated PDA*, *n***	**6**
*Age*, *median (range)*, *y*	61 (58–61)
*Male*, *n (%)*	3 (50%)
*Female*, *n (%)*	3 (50%)
***Premalignant*, *n***	**13**
*Age*, *median (range)*, *y*	58 (28–79)
*Male*, *n (%)*	5 (38%)
*Female*, *n (%)*	8 (62%)
***Benign*, *n***	**10**
*Age*, *median (range)*, *y*	53 (40–71)
*Male*, *n (%)*	5 (50%)
*Female*, *n (%)*	5 (50%)
***Neuroendocrine*, *n***	**3**
*Age*, *median (range)*, *y*	61 (55–69)
*Male*, *n (%)*	2 (66%)
*Female*, *n (%)*	1 (33%)
***Duodenal Adeonocarcinoma*, *n***	**1**
*Age*, *median (range)*, *y*	61
*Male*, *n (%)*	1 (100%)
*Female*, *n (%)*	0 (0%)

### RNASeq analysis of pancreatic cancer

Of the patients above with pancreatic masses, we randomly selected 39 patients with pathologically diagnosed pancreatic adenocarcinoma with no neoadjuvant treatment and with matched quantitative IHC for RNASeq. All subsequent analyses of IHC and RNASeq were performed on unpretreated patients. A representative Hematoxylin and Eosin (H&E) stained slide for each formalin-fixed paraffin-embedded (FFPE) tissue block specimen was reviewed by a board-certified pathologist for tumor content and tumor-rich regions were identified for microdissection. Blocks were matched but not in series with IHC sections. 5 μm thick unstained sections on glass slides were processed for DNA and RNA purification by the Providence Molecular Genomics Laboratory. The FFPE tissue sections were deparaffinized using Envirene (Hardy Diagnostics) followed by RNA extraction and purification using the Qiagen AllPrep DNA/RNA FFPE kit. 85ng of input RNA was used to prepare sequencing libraries using the Illumina TruSeq RNA Exome kit. Sequencing of the RNA Exome libraries was performed on the Illumina HiSeq 4000 instrument at 2 x 75 read paired end configuration. Transcripts were quantified using salmon-v.0.11.2 [9]. A matrix of gene expression values for all patients analyzed in this study along with matched quantitative IHC are provided as a S1 Table.

### Computational analysis of infiltrating cells and comparison of techniques

RNA-Seq-based cell type deconvolution was performed using xCELL (5) and CIBERSORT (6), with TPM gene expression levels as input. xCELL was used to perform *cell type enrichment analysis* from gene expression data for 64 immune and stroma cell types, whereas CIBERSORT provides *absolute and relative abundance* of different immune cell types depending on the specified gene set. In the present analysis, the LM22 signature provided by CIBERSORT was applied. We also applied EPIC (10) and MCPcounter (11) to estimate the abundance of immune cell population. EPIC estimates the proportions of Immune and Cancer cells by using RNA-Seq-based gene expression reference profiles from immune cells and other nonmalignant cell types found in tumors. MCPcounter quantifies abundance of tissue-infiltrating eight immune and two stromal cell populations based on transcriptome profile.

Initial clustering of patients based on infiltrating cell types was performed using ClusterVis [10]. Principal components are calculated as described in (6) using ClusterVis (7). Missing data is assigned using Singular Value Decomposition with imputation iteratively until estimates of missing values converge. Statistical significance of the resulting hierarchical clusters were assessed using the sigclust2 R package [11]. Correlation between infiltrating cell types calculated using RNA-Seq versus quantitative IHC was performed by generating a composite of T cell and macrophage cell types determined by RNA-Seq for direct comparison to IHC populations (Table 2). Correlations between xCELL immune cell type enrichment and quantitative IHC, as well as between CIBERSORT cell type abundances and quantitative IHC, were determined by Spearman Rank correlation.

**Table 2:** Assembly of composite RNASeq-calculated populations to match cell types detected by IHC.

IHC populations	CD3		CD8	CD68
*CIBERSORT*	T.cells.CD8	T.cells.CD4.naive	T.cells.CD8	Macrophages.M0
	T.cells.CD4.memory.resting			Macrophages.M1
	T.cells.CD4.memory.activated	T.cells.follicular.helper		Macrophages.M2
	T.cells.regulatory.Tregs	T.cells.gamma.delta		
*xCELL*	CD4+ memory T-cells	CD4+ naive T-cells	CD8+ naive T-cells CD8+ T-cells CD8+ Tcm CD8+ Tem	Macrophages
	CD4+ T-cells	CD4+ Tcm		Macrophages M1
	CD4+ Tem	CD8+ naive T-cells		Macrophages M2
	CD8+ T-cells	CD8+ Tcm		
	CD8+ Tem	Tregs		
	Th1 cells	Th2 cells		
	Tgd cells	NKT		

### Identification of genes associated with high and low T cell infiltration

Patients were categorized into high and low infiltration of CD3^+^ and CD8^+^ T cells according to quantitative IHC and sub-categorized into those with high infiltration of both populations (CD3^HI^CD8^HI^) versus low infiltration of both populations (CD3^LO^CD8^LO^). Using RNA-Seq data from these patients, gene expression analyses were performed using a univariate two-sample T-test with a stringent false-positive threshold to identify genes significantly differentially expressed (p<0.001) in our patients [12, 13]. These genes were mapped to known pathways using the Reactome Functional Interaction network tool [14]. These genes were tested on the TCGA Pancreatic Adenocarcinoma PanCancer Atlas [15] as a validation cohort using cBioportal [16, 17] where mRNA expression z-scores are compared to the expression distribution of each gene in tumors that are diploid for this gene. To be classified as enriched for the gene score the sample must have at least one gene that is 2 log fold overexpressed. Survival and expression data exported to Graphpad Prism for survival comparison using log-rank tests. Pre-calculated xCELL analysis of patients in the TCGA database were obtained from http://xcell.ucsf.edu.

### Statistical methods

Survival data were analyzed using Prism (Version 8.4.2, GraphPad Software, La Jolla, CA). Overall survival of groups was compared using a log rank test for differences in Kaplan-Meier survival curves. All cutoffs for high/low infiltration by RNA analysis use median values. Gene expression analyses were performed using a univariate two-sample T-test with a stringent false-positive threshold to identify genes significantly differentially expressed (p<0.001) [12, 13]. Correlations between immune cell type enrichment and quantitative IHC were determined by Spearman Rank correlation. To assess the statistical significance of correlation between RNA-Seq-based cell type deconvolution and CD3, CD8 or CD68 immune cell types determined by quantitative IHC, the composition of underlying cell types specified in Table 2 were randomly permuted. The number of cells that constitute a cell type were kept the same between the true set and permuted set. Owing to the number of cell types estimated by xCELL (64 different cell type populations) and CIBERSORT (22 different cell type populations), we conducted 100 and 20 different random assignments of cell types, respectively, attributed to the CD3, CD8 and CD68 IHC populations in Table 2. For each permutation, Spearman rank correlation was computed between the random cell type assignments and quantitative IHC, allowing for a level of statistical significance to be estimated for the true set with respect to all permuted sets, thereby indicating whether the RNA-Seq-based cell type deconvolution methods are statistically significantly correlated with quantitative IHC. Additional correlations between multiple variables were analyzed using Prism (Version 8.4.2) to calculate a Pearson correlation coefficient. Statistical significance of hierarchical clusters were assessed using the sigclust2 R package [11]. Differential gene expression analysis between CD3 Hi CD8 HI vs CD3 Lo CD8 Lo RNA-Seq samples were carried out using DESeq2 [18]. We identified differentially expressed genes, ranked them based on fold-change and p-value. This gene signature was further used for gene set enrichment.

## Results

**Fig 1:**
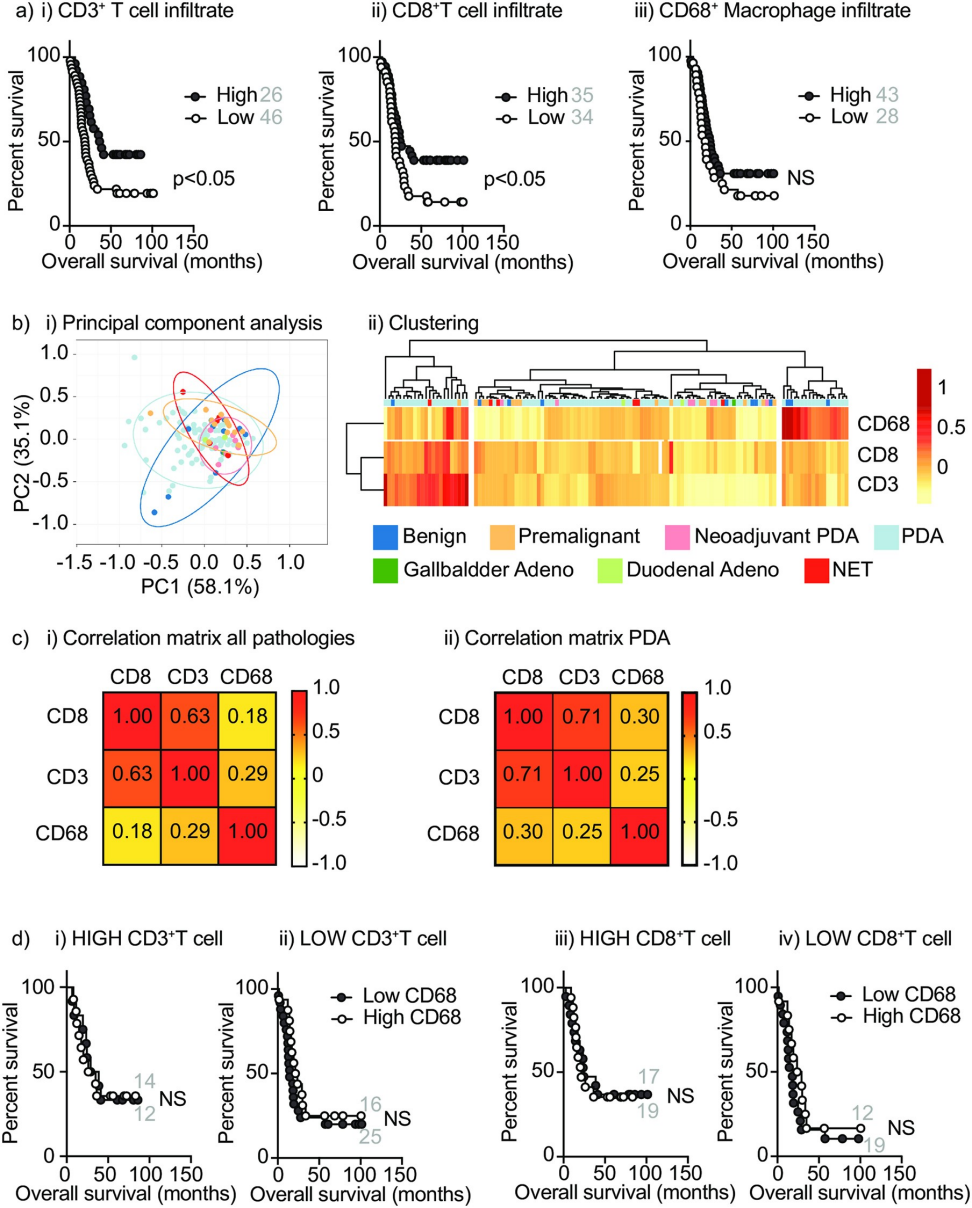
Analysis of patients by quantitative immunohistology. a) Quantitative analysis of infiltrating i) CD3^+^; ii) CD8^+^; and iii) CD68^+^ cells/mm^2^ tumor were used to set cutoff values of high/low infiltrates with significant impact on overall survival of patients with PDA. Cutoffs used were approximately CD3 – 75^th^ percentile; CD8 –median; CD68 –no significant cutoff found, 75^th^ percentile shown. b) Infiltrating CD8^+^, CD3^+^, and CD68^+^ cells across a range of pathologies was used to evaluate principal component analysis and clustering. i) Unit variance scaling is applied to rows; SVD with imputation is used to calculate principal components. X and Y axis show principal component 1 and principal component 2 that explain 56.7% and 30.5% of the total variance, respectively. Prediction ellipses are such that with probability 0.95, a new observation from the same group will fall inside the ellipse. N = 123 data points. ii) Clustering of patients according to infiltrates. Imputation is used for missing value estimation. Both rows and columns are clustered using Manhattan distance and complete linkage. 3 rows, 123 columns. c) Pearson correlation coefficients for CD8, CD3 and CD68 infiltrating cells in i) all pathologies; ii) PDA. d) Overall survival of PDA patients with i) high or ii) low CD3^+^ infiltrates, and iii) high or iv) low CD8^+^ infiltrates subdivided according to high or low CD68 infiltrates as determined in a). Number of patients on each arm of survival curves are shown in grey.

We previously demonstrated that increased CD3^+^ T cell infiltrates in surgically resected pancreatic adenocarcinoma correlate with improved outcomes using a Cox proportional hazards model, and remained prognostic by multivariable analysis [1]. By contrast, CD8^+^ T cell infiltrates and CD68^+^ macrophage infiltrates did not correlate with outcome [1]. However, using categorical variables of high or low CD3^+^, CD8^+^ and CD68^+^ cell numbers in tumors, we were able to identify cutoffs that demonstrated that patients with high CD3^+^ T cells or high CD8^+^ T cells exhibited improved survival, but again CD68^+^ macrophages did not correlate with survival (Fig 1a). To evaluate the complexity of the infiltrate in patient tumors, we performed hierarchical cluster analysis. Initially, we used our broader dataset that included pancreatic ductal adenocarcinoma (PDA) with and without neoadjuvant treatment, as well as benign pancreatic masses, pre-malignant disease, neuroendocrine tumors, as well as a small number of duodenal adenocarcinoma and gallbladder adenocarcinoma. Principal component analysis was not able to distinguish these pathologies based on CD3^+^, CD8^+^ and CD68^+^ cell infiltrate (Fig 1bi), and while cluster analysis tended to group benign and premalignant disease in poorly infiltrated groups, there was not a clear classifier to distinguish PDA from other related pathologies (Fig 1bii). Prior studies have demonstrated that patients with the highest macrophage proportions and lowest CD8^+^ T cell proportions exhibit worse outcome than those with lowest macrophage proportions and highest T cell proportions [19]. We calculated the correlation between CD3^+^, CD8^+^ and CD68^+^ cell infiltrate in patients with all pathologies and those with PDA and found good correlation between CD3^+^ and CD8^+^ infiltrates, and poor, but not negative, correlation with T cells and CD68^+^ cell infiltrate (Fig 1c). To determine whether the degree of CD68^+^ cell infiltrate impacted outcome for patients with high or low T cell infiltrates, we tested the effect of CD68^+^ cell infiltrate with each T cell cutoff. For patients with PDA we did not find an effect of macrophage infiltration on the survival benefit of CD3^+^ T cells (Fig 1di and 1dii) or CD8^+^ T cells (Fig 1diii and 1div). These data demonstrate that quantitative immunohistology was able to identify good and poor outcome groups based on CD3^+^ and CD8^+^ T cell infiltrate, but analyzing the degree of CD68^+^ macrophage infiltrate alone or in combination with T cell infiltrates did not help refine outcome groups.

**Fig 2:**
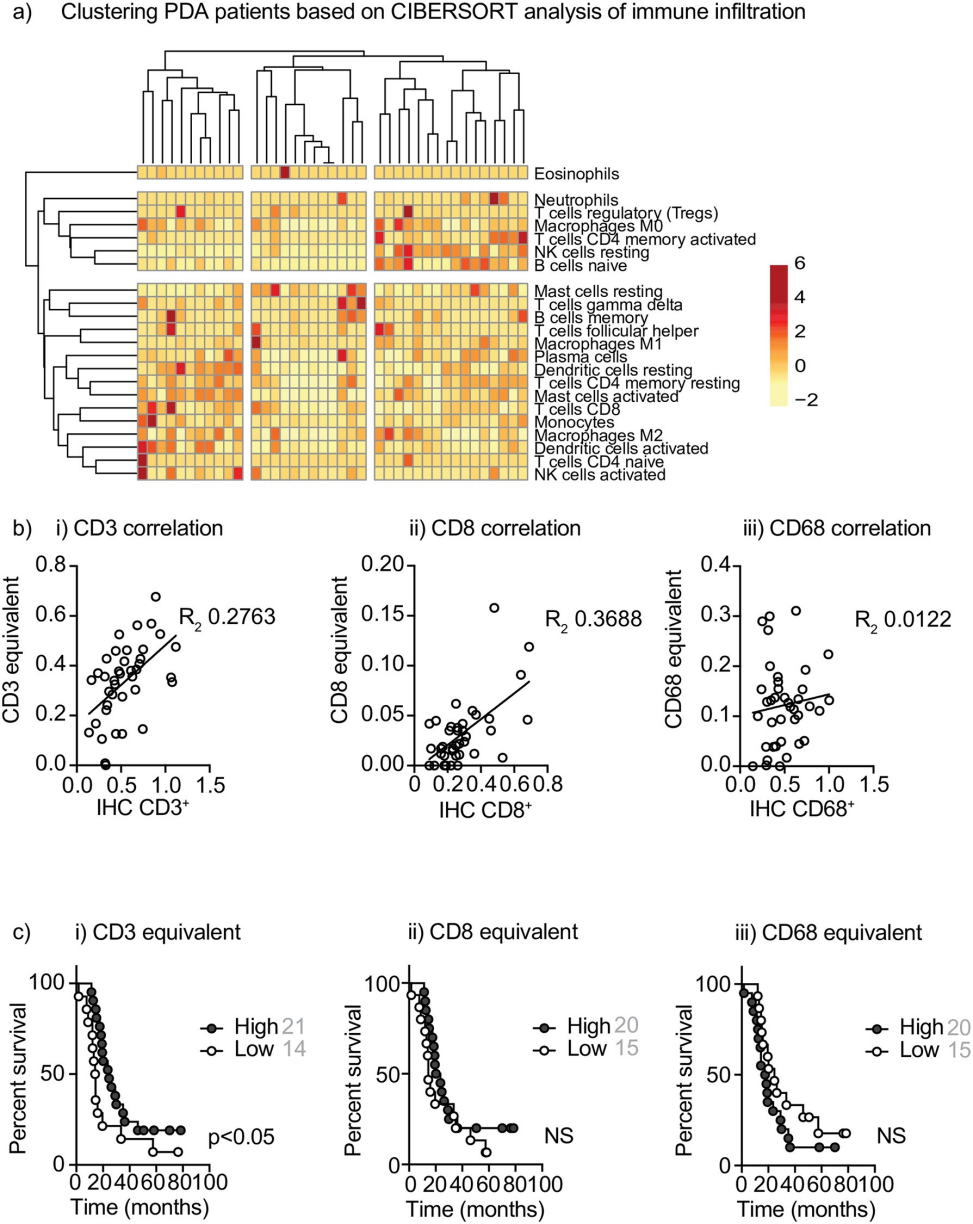
Evaluation of CIBERSORT analysis of immune infiltration. a) Clustering of immune populations inferred using CIBERSORT analysis of RNASeq of PDA patients. Rows are centered; unit variance scaling is applied to rows. Both rows and columns are clustered using correlation distance and average linkage. 22 rows, 39 columns. b) The immune infiltrate score of all i) CD3^+^, ii) CD8^+^, and iii) CD68^+^ equivalent cell populations (Table 2) determined by CIBERSORT compared to quantitative IHC from the same patient. Each symbol represents one patient. c) Overall survival of patients with high versus low infiltrates of i) CD3^+^, ii) CD8^+^, and iii) CD68^+^ equivalent cell populations (Table 2) determined by median CIBERSORT infiltration. NS = not significant.

There are significant limitations in the use of CD68 as a sole marker of macrophages in tumors [20], particularly in view of the diverging phenotypes macrophages can generate. There is not a well-defined set of markers that is unique to distinct macrophage polarization states that do not overlap with other cell types. Recently, a number of algorithms have been developed that can analyze gene expression data to estimate the prevalence of the broad range of cell types in a mixed tissue sample [5, 6, 21]. To evaluate whether gene expression analysis could refine our understanding of the immune environment of pancreatic cancer we tested two different approaches, CIBERSORT [6] and xCELL [5]. CIBERSORT has been most widely applied, and provides an assessment of 22 of the most common immune cell types and some information on the differentiation of CD4^+^ T cells and macrophages [6]. We performed RNA-Seq on a subset of our PDA patients with quantitative IHC, and performed CIBERSORT analysis of immune infiltration using the RNA-Seq data. We used a correlation analysis to determine whether some cell types were co-regulated in the tumor, but there was little evidence of correlation between the infiltration of different immune cell types in the tumor (S1 Fig). We performed cluster analysis on patients based on their immune infiltrates calculated by CIBERSORT (Fig 2ai), which appeared to identify a diffuse cluster of patients with higher numbers of CD8 T cells and dendritic cells, but there was no strong statistical association between these cell types and this analysis was not able to identify patient groupings with significant differences in overall survival (not shown). To directly compare these CIBERSORT calculated infiltrating cell types to the quantified immunohistology from the same samples, we made three combined groups (Table 2): 1. CD3^+^ equivalent based on cell types that express CD3 (Treg+CD4 populations+CD8+gd T cells); CD8^+^ equivalent (CD8); and CD68^+^ macrophage equivalent (MO+M1+M2). We then evaluated the correlation between infiltration determined by CIBERSORT analysis of RNA-Seq versus quantified immunohistology. Both CD3^+^ and CD8^+^ T cells showed a weakly positive correlation between the IHC and CIBERSORT assessments, but CD68 did not correlate well between histology and CIBERSORT (Fig 2b). Spearman’s rho computed between IHC and CIBERSORT relative cell type abundance were 0.355, 0.308, 0.144 for CD3, CD8 and CD68 respectively. Notably, we see a number of patients with no detectable infiltrating T cells or macrophages by CIBERSORT, who did have detectable cells by histology. Since CIBERSORT is dependent on key RNA transcripts being present amongst the RNA sequenced, we believe that at low cell infiltration this approach can struggle to identify the RNA signature of rare infiltrating cells. To determine whether CIBERSORT infiltration of these key cell types predicted outcome, we similarly stratified patients into high or low infiltration groups. We found that patients with high combined CD3^+^ equivalent scores exhibited improved overall survival (p<0.05), but the CD8 and macrophage scores were not able to discriminate patients with significantly different overall survival (Fig 2c). These suggest that while CIBERSORT analysis can provide additional information on the diversity of immune cells in tumors, it does not improve our ability to predict outcome over quantitative histology. Since there is a general agreement between the assessment of total CD3 infiltrate by histology and CIBERSORT and each are associated with improved outcome in patients, aggregating CIBERSORT T cell infiltration could be further tested as a prognostic factor in pancreatic cancer patients.

**Fig 3:**
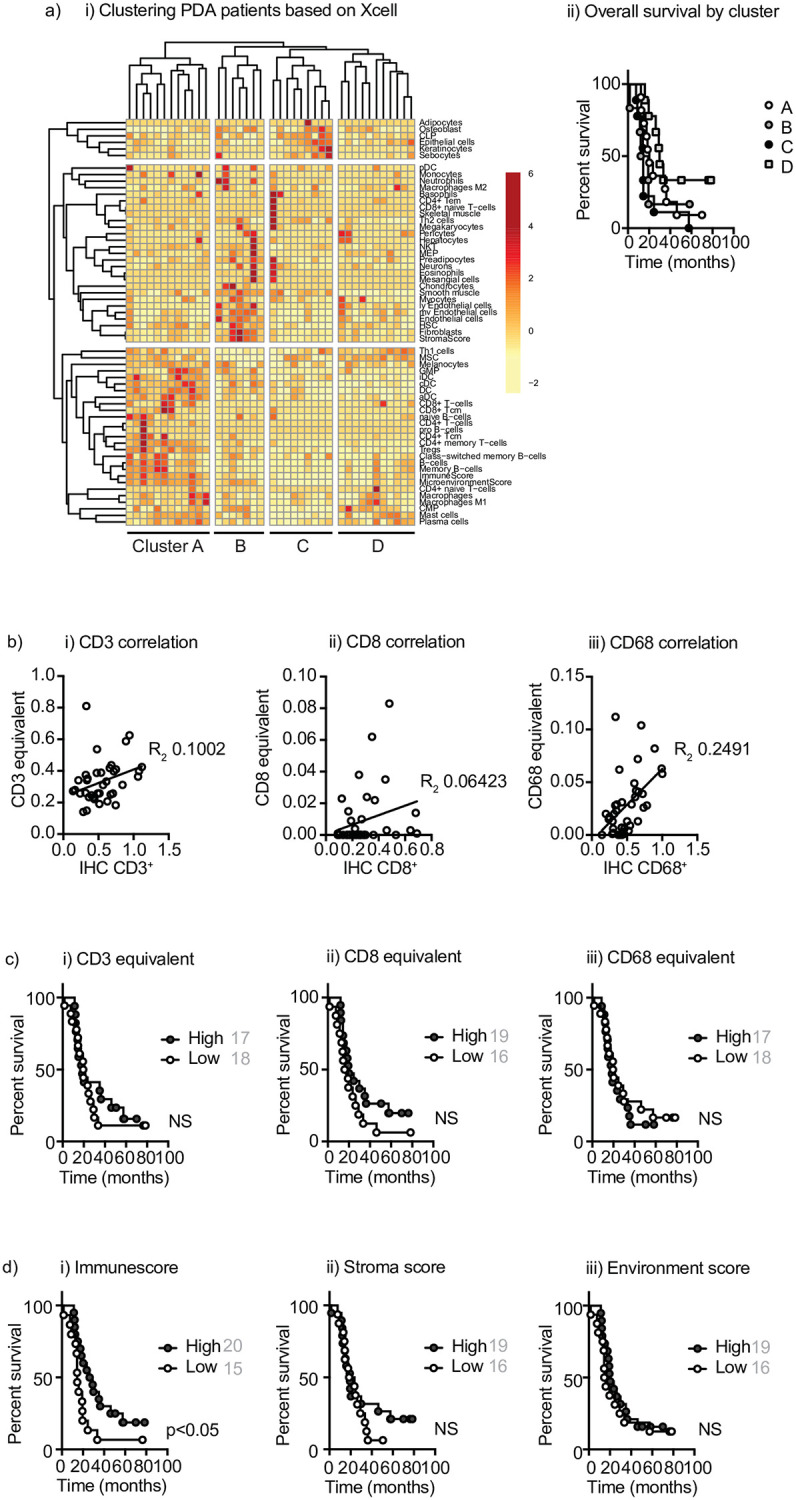
Evaluation of xCELL analysis of immune infiltration. a) i) Clustering of immune populations inferred using xCELL analysis of RNASeq of PDA patients. Rows are centered; unit variance scaling is applied to rows. Both rows and columns are clustered using correlation distance and average linkage. 60 rows, 39 columns. ii) overall survival of patients in clusters A, B, C, and D. b) The immune infiltrate score of all i) CD3^+^, ii) CD8^+^, and iii) CD68^+^ equivalent cell populations (Table 2) determined by xCELL compared to quantitative IHC from the same patient. Each symbol represents one patient. c) Overall survival of patients with high versus low infiltrates of i) CD3^+^, ii) CD8^+^, and iii) CD68^+^ equivalent cell populations (Table 2) determined by median xCELL infiltration. NS = not significant. d) Overall survival of patients with high versus low i) immunescore, ii) stromascore, and iii) environment score using median values as cutoffs.

As an alternative approach xCELL can identify 64 different cell populations and composite infiltration scores from RNA-Seq data [5]. We performed xCELL analysis of immune infiltration in pancreatic cancer and generated a correlation matrix to examine associations between different cell types. Clear patterns emerged, with some tight clusters based around epithelial cells or Th2 cells, and more broad groupings of co-regulated cells including Th1 cells, DC, M1 macrophages and CD8 T cells (S2 Fig). To determine whether these co-resident cells identified unique patient populations, we clustered patients based on their immune infiltrate, identifying patients with higher levels of CD8 T cells, DC, and M1 macrophages (Cluster A), and those with higher levels of fibroblasts and endothelial cells to form a distinct cluster (Cluster B) (Fig 3a). Comparing the overall survival of patients in each cluster demonstrated there were no significant differences between groups (Fig 3b). In view of the high correlation between specific cell types resulting in apparent clusters, we investigated whether this was due to closely related cells having overlapping genes that are included in the underlying gene signature. Using the gene signatures that determine cell types in xCELL, we computed the percent match between genes across all cell types (S3 Fig). These data demonstrated that most cells were defined using a unique gene set. We did identify overlapping gene usage between related cell types such as DC subtypes or T cell subtypes; however, there were no significant gene overlaps between DC and T cells, for example, that would explain their correlation. These suggest that the positive correlation between the number of T cells and DCs in patient tumors is likely a result of the presence of both cell types in the analyzed sample. To compare these calculated infiltrating cell types to quantified immunohistology, we again made three combined groups: 1. CD3 equivalent (Treg + all CD4 populations + all CD8 populations +gd T cells); CD8 equivalent (all CD8 populations); and CD68 macrophage equivalent (MO + M1 + M2) (Table 2). We evaluated the correlation between cell infiltration assayed by xCELL analysis of RNA-Seq versus quantified immunohistology. Each population showed a positive correlation between the two approaches; however, many of the samples were calculated to have no CD8 T cells by xCELL, even in patients with relatively abundant CD8 T cells as determined by immunohistology (Fig 3b). As with CIBERSORT analysis, despite the positive correlation, the R^2^ value was not strong for each cell type. Spearman’s rho computed between IHC and xCELL were 0.354, 0.307 and 0.144 for CD3, CD8 and CD68 respectively. We also evaluated whether infiltration of these cell types impacted outcome, and we could not detect a cutoff that impacted overall survival (Fig 3c).

xCELL analysis does calculate three additional fields that integrate many of the infiltrating cell features to generate an “immunescore”, a “microenvironment score” and a “stromascore”. Such combined fields may have an advantage over individual cell types, particularly where the cells are of low abundance. Broadly, the immunescore was correlated with T cell infiltration, while the stromascore was correlated with fibroblast and endothelial cell infiltration (S3 Fig). Patients with a high immunescore exhibited improved overall survival, but the stromascore and the microenvironment score were not able to distinguish patient groups with improved outcome. These data suggest that xCELL can provide a more complex understanding of the immune cell diversity in tumors; however, there remain significant issues identifying the small numbers of T cells infiltrating pancreatic adenocarcinoma. There may be a benefit in integrating multiple immune cell types through features such as the immunescore to identify tumor environments indicative of improved outcome.

There are increasing numbers of methods to analyze cell infiltrates from RNA data. We compared the additional methods EPIC [22] and MCPcounter [23] with the same dataset. The MCPcounter assessment of T cell infiltration correlated well with the IHC CD3 infiltrate, but all other analyses had poor correlation to IHC data (S4 Fig). To understand whether there was agreement amongst the various RNA-based approaches, we analyzed the correlation between the various infiltrating T cell populations assessed by CIBERSORT, xCELL, MCPcounter, and EPIC. The correlation between the different approaches using the same RNA dataset was moderate, but the closest correlations were found among CD4 T cell populations and relatively poor correlations between CD8 T cell populations (S4 Fig). These data suggest that there are significant differences between the RNA-based approaches and each has difficulty consistently identifying CD8 T cell infiltrates in T cell poor tumors like pancreatic cancer.

**Fig 4:**
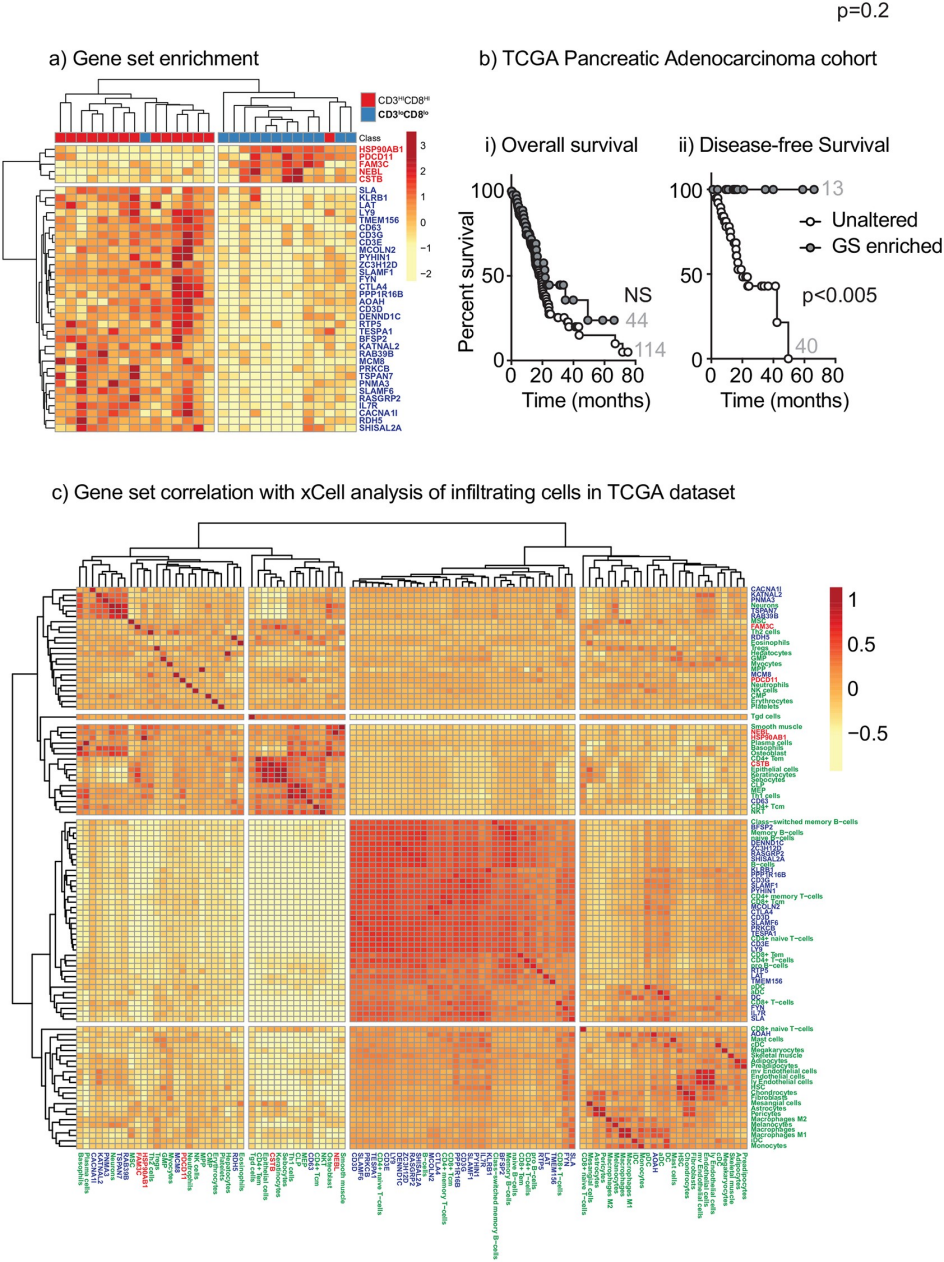
Class comparison of TPM counts on patients with high T cell infiltrate and validation on TCGA cohort. a) Class comparison of TPM counts was performed on patients with high CD3^+^ and high CD8^+^ infiltrates, as compared to patients with low CD3^+^ and low CD8^+^ infiltrates using the cutoffs applied in Fig 1. Graph shows the clustering of 38 significant genes (p<0.001). Genes enriched in poorly infiltrated tumors are highlighted in bold. b) The genes enriched in highly infiltrated tumors were tested using the TCGA PanCan Pancreatic Adenocarcinoma cohort to identify patients with enriched RNA expression of these genes. Graphs show i) overall survival and ii) disease-free survival for patients in each group. The number of patients in each group is shown. c) Correlation matrix of the expression of the genes listed in a), and xCELL calculated cell infiltration for the TCGA PanCan Pancreatic Adenocarcinoma cohort. Rows are centered; no scaling is applied to rows. Both rows and columns are clustered using Manhattan distance and average linkage. 102 rows, 102 columns. Genes are highlighted in Blue where positively correlated and Red when negatively correlated. Cell types are highlighted in Green.

To determine whether there is an alternative RNA signature of high T cell infiltration that can be used in pancreatic tumors to infer T cell infiltration and assess outcome using RNA-Seq samples, we identified genes that were enriched in tumors with both high CD3 and high CD8 infiltration or both low CD3 and low CD8 infiltration by quantitative IHC. Class comparison of gene expression identified a subset of genes that were statistically associated with highly T cell infiltrated tumors (Table 3, Fig 4a). As would be predicted, these included genes encoding for CD3 as well as genes involved in T cell signaling such as FYN and LAT. Interestingly, the gene set includes the immunotherapy target CTLA4 [24], as well as SLAMF6, which is a marker of progenitor exhausted T cells [25]. To determine whether increased expression of these genes were useful predictors of outcome in pancreatic cancer patients, we examined expression of these genes in pancreatic adenocarcinoma patients in the TCGA database [15], and their effect on patient outcome. Initial analysis indicated that patients with increased expression of the genes positively associated with T cell infiltration in our cohort had significantly increased overall survival and disease-free survival. However, following curation of the TCGA dataset according to Peran *et al*. [26] to remove mischaracterized tumors from the cohort, overall survival was no longer significantly different, but disease-free survival was significantly improved (Fig 4b). These data suggest that the geneset was identifying patients with neuroendocrine tumors and the improved prognosis of these patients was influencing the overall survival results in the uncurated dataset. To understand whether the geneset was associated with increased T cell infiltration, we obtained pre-calculated xCELL analysis of infiltrating cells in these pancreatic cancer TCGA specimens (https://xcell.ucsf.edu), and examined the correlation of each cell type with the expression of genes in our panel. To find potential patterns of biological significance, we correlated the expression of the gene set with the infiltration of immune cells across the TCGA cohort and performed clustering to gather co-regulated genes and cells together. We discovered that as with our cohort, there was a strong correlation between infiltrating T cells and dendritic cells in pancreatic tumors, and these correlated closely with the expression of genes in our panel (Fig 4c). Macrophage and endothelial cell infiltration did not correlate well with any of the genes in our panel, and smooth muscle cells, keratinocytes, and epithelial cell infiltration correlated best with some of the genes that were negatively associated with T cell infiltration, such as NEBL and HSP90AB1. These data indicate that the gene signature associated with high T cell infiltration in our pancreatic cancer cohort can similarly identify high T cell infiltration in other pancreatic cancer cohorts represented in the TCGA database. Further studies are needed to understand whether these genes have functional roles and are potential therapeutic targets.

**Table 3:** Normalized log transformed expression of genes that are statistically associated with highly T cell infiltrated tumors.

*Class*	*HI*	*HI*	*HI*	*HI*	*HI*	*HI*	*HI*	*HI*	*HI*	*HI*	*HI*	*HI*	*HI*	*HI*	*HI*	*LOW*	*LOW*	*LOW*	*LOW*	*LOW*	*LOW*	*LOW*	*LOW*	*LOW*	*LOW*	*LOW*	*LOW*	*LOW*	*TTest* *pvalue*	*DESeq2* *pvalue*
***IL7R***	33.09	31.01	49.75	47.16	35.12	46.06	33.44	52.94	61.96	18.56	27.38	43.68	61.44	24.79	46.79	14.89	31.59	11.41	9.17	18.18	19.15	22.71	18.08	25.04	17.17	19.97	5.41	10.07	**3.70E-06**	**0.000001**
***TESPA1***	1.24	1.81	2.72	2.00	1.82	2.75	1.85	1.58	1.31	1.78	2.34	1.23	2.39	0.85	1.43	0.56	1.89	0.14	0.25	0.76	0.00	0.92	0.00	0.00	0.56	1.58	0.00	0.25	**5.00E-06**	**0.010905**
***SLAMF1***	1.71	1.66	1.86	1.53	1.94	2.05	1.86	2.00	1.87	1.99	1.24	2.00	2.27	1.17	1.48	0.53	1.88	0.64	0.47	1.63	1.17	1.27	0.00	0.00	1.00	1.09	0.17	0.73	**9.00E-06**	**0.023359**
***KATNAL2***	1.16	1.10	0.80	1.10	0.54	1.59	0.53	1.33	1.74	1.49	0.94	1.27	0.89	0.64	0.77	0.22	0.70	0.70	0.35	0.17	0.28	0.64	0.00	0.00	0.72	0.37	0.58	0.23	**9.90E-06**	**0.177889**
***BFSP2***	2.26	4.55	3.80	3.58	3.28	7.70	5.61	3.66	4.81	1.91	2.48	4.17	3.53	1.81	3.81	1.69	3.07	0.93	0.27	1.47	2.03	1.14	1.28	0.00	1.40	2.61	0.69	0.21	**2.06E-05**	**0.875888**
***SLAMF6***	2.30	3.71	4.72	3.62	3.11	3.82	4.35	3.44	6.08	4.47	2.04	5.56	5.27	2.21	5.15	2.14	3.88	0.33	0.72	2.35	2.37	2.87	0.00	1.78	1.13	2.36	0.67	1.30	**2.44E-05**	**0.000220**
***MCOLN2***	2.15	2.12	1.54	2.45	2.06	4.15	0.59	1.89	3.11	3.13	2.20	2.49	4.44	2.12	2.33	1.35	2.00	0.26	0.48	0.97	2.21	0.84	0.50	0.42	0.83	0.95	0.42	0.42	**3.27E-05**	**0.000000**
***PRKCB***	0.69	0.92	0.91	1.25	1.31	1.16	0.37	1.27	2.25	1.04	0.47	1.23	1.19	1.08	2.11	0.48	0.68	0.26	0.26	0.78	0.48	0.56	0.14	0.00	0.65	0.32	0.13	0.86	**0.0001019**	**0.000003**
***RDH5***	2.77	2.88	4.16	3.64	3.69	2.45	5.33	3.09	11.45	4.73	2.53	5.77	5.09	2.68	3.50	0.01	2.45	0.02	0.01	2.58	0.03	4.77	0.00	0.00	1.84	0.52	0.00	0.01	**0.0001349**	**0.006895**
***CACNA1I***	0.12	0.44	0.75	0.43	0.55	0.68	0.42	0.58	0.71	0.32	0.00	0.58	0.99	0.22	0.45	0.14	0.07	0.09	0.03	0.19	0.00	0.43	0.44	0.00	0.00	0.23	0.00	0.03	**0.000179**	**0.000292**
***FAM3C***	12.91	1.59	9.15	11.56	19.41	3.64	11.79	4.44	11.84	9.69	4.43	18.49	3.51	5.53	22.41	5.16	20.21	26.98	28.42	4.07	29.48	42.83	34.86	37.29	11.78	33.21	25.53	31.85	**0.0001943**	**0.000008**
***RASGRP2***	7.43	10.49	12.13	8.82	9.39	12.43	8.48	12.29	11.81	9.22	4.61	10.41	14.91	3.99	21.65	4.45	8.13	0.94	3.61	8.81	4.37	10.78	0.00	0.00	4.99	6.11	0.89	1.92	**0.0002413**	**0.000015**
***PNMA3***	0.82	1.35	1.98	0.98	0.67	0.84	0.25	1.18	1.90	0.48	0.65	0.70	1.27	0.29	1.17	0.35	0.63	0.13	0.11	0.54	0.25	0.39	0.00	0.00	0.38	0.87	0.13	0.21	**0.0002839**	**0.001358**
***CD3G***	12.32	8.93	12.44	10.73	14.99	13.95	9.65	10.06	6.52	13.96	6.25	13.48	20.85	12.03	14.86	4.68	11.84	2.87	2.96	6.93	8.67	9.90	0.34	9.61	9.85	7.97	1.90	3.81	**0.0002999**	**0.000249**
***CD3D***	55.87	27.13	40.82	58.87	38.77	73.28	42.79	42.60	20.33	69.71	24.77	48.64	86.30	35.96	64.84	17.99	50.76	7.99	12.86	26.56	41.04	40.76	0.00	0.00	29.82	20.72	3.06	14.31	**0.0003143**	**0.001063**
***DENND1C***	5.97	4.88	4.82	5.38	6.90	8.00	4.47	4.03	2.94	6.38	5.58	5.53	7.93	4.05	6.45	2.76	5.23	2.59	1.83	2.82	4.21	5.73	0.00	0.00	4.12	5.19	0.22	2.83	**0.0003418**	**0.000066**
***CD3E***	40.56	42.42	56.30	45.07	63.07	56.70	35.61	39.14	40.75	44.51	22.08	44.56	88.82	35.33	61.43	18.52	48.81	14.52	11.04	19.37	35.77	34.25	16.35	46.24	29.72	29.81	5.68	12.46	**0.0003708**	**0.000016**
***AOAH***	7.84	2.54	8.64	3.57	9.02	11.51	6.07	7.73	2.87	9.80	6.54	1.85	14.34	8.12	8.93	1.97	5.88	1.87	1.57	4.62	3.22	3.83	0.00	0.00	5.58	4.92	1.69	2.04	**0.0004128**	**0.007445**
***TSPAN7***	14.92	24.09	11.61	14.69	14.14	11.60	6.33	13.84	20.89	10.25	5.60	12.87	12.43	5.92	26.52	8.01	8.85	4.27	3.15	9.63	3.12	7.19	0.00	5.21	13.40	7.05	5.93	1.91	**0.0004985**	**0.000764**
***KLRB1***	10.77	26.09	23.84	17.58	18.70	16.56	18.88	22.95	15.47	20.01	16.00	24.41	25.87	19.44	32.76	12.52	13.52	6.09	9.01	12.74	12.86	16.24	28.90	0.00	14.34	9.53	3.59	6.89	**0.0005089**	**0.000001**
***LY9***	1.25	0.27	0.40	0.24	2.21	2.56	1.03	1.89	0.00	0.00	1.71	0.82	2.42	1.77	2.76	0.00	0.70	0.08	0.00	0.27	0.67	0.00	0.00	0.00	0.57	0.00	0.12	0.00	**0.0005254**	**0.389805**
***ZC3H12D***	0.32	0.41	0.41	0.35	0.53	0.78	0.43	0.44	0.48	0.65	0.35	0.40	0.45	0.37	0.47	0.31	0.59	0.26	0.16	0.39	0.35	0.34	0.00	0.13	0.30	0.19	0.08	0.15	**0.0005471**	**0.001851**
***FYN***	10.68	9.05	9.98	8.94	15.26	20.90	11.46	13.68	11.13	9.11	8.95	10.38	16.12	9.10	13.27	4.15	11.56	5.14	6.29	11.16	11.07	9.60	0.00	0.00	7.04	8.89	2.04	4.97	**0.0005492**	**0.063184**
***LAT***	1.39	2.75	1.73	1.43	2.38	2.09	3.71	2.96	2.52	4.01	1.37	1.83	2.93	1.63	3.34	1.05	1.81	1.19	0.97	1.53	2.16	2.07	1.97	0.33	1.38	1.14	0.23	0.51	**0.0005681**	**0.683191**
***NEBL***	3.48	1.63	3.90	2.55	3.35	2.50	2.15	2.65	1.78	3.47	2.24	4.16	6.73	1.76	3.11	3.47	5.87	6.01	5.56	6.16	8.67	4.12	12.11	9.66	3.75	5.45	2.67	13.52	**0.0005814**	**0.000026**
***HSP90AB1***	221.53	353.00	258.65	266.09	307.87	268.80	268.16	201.39	0.02	153.03	395.19	260.95	228.47	165.12	198.69	149.89	276.35	481.85	551.54	378.17	433.15	494.92	481.83	458.89	365.47	487.20	164.87	469.71	**0.0006012**	**0.028536**
***RTP5***	0.03	0.07	0.03	0.08	0.06	0.14	0.14	0.07	0.09	0.11	0.02	0.01	0.15	0.03	0.05	0.03	0.02	0.00	0.06	0.07	0.00	0.02	0.00	0.00	0.01	0.03	0.00	0.00	**0.0006258**	**0.002330**
***RAB39B***	0.23	0.19	0.20	0.34	0.15	0.28	0.05	0.26	0.31	0.30	0.16	0.65	0.18	0.20	0.29	0.14	0.22	0.09	0.02	0.10	0.18	0.15	0.00	0.00	0.10	0.03	0.10	0.08	**0.0007029**	**0.001085**
***TMEM156***	2.94	4.66	3.70	3.58	5.42	5.96	3.42	4.31	0.00	2.49	1.54	5.45	7.60	3.93	6.00	1.73	4.27	2.36	1.83	2.00	1.93	0.45	0.00	0.86	3.51	1.17	0.43	1.67	**0.0007365**	**0.026697**
***MCM8***	0.21	9.08	2.47	4.87	2.89	3.68	4.92	0.72	7.11	2.36	0.30	5.56	6.33	1.31	4.26	0.00	3.32	1.07	0.00	0.00	3.09	1.34	0.00	0.00	0.37	0.16	0.15	0.00	**0.0007729**	**0.807953**
***CTLA4***	4.26	5.21	6.41	5.36	9.10	10.90	3.29	5.17	4.14	4.44	4.08	5.06	10.16	4.95	4.81	2.13	8.82	1.69	0.69	2.35	4.03	3.04	0.00	0.00	3.65	2.12	0.53	3.05	**0.0007741**	**0.000770**
***CSTB***	350.66	213.93	172.57	375.21	241.66	255.96	149.99	214.06	112.22	154.54	275.36	214.92	173.51	185.79	258.47	194.88	393.89	315.94	423.05	484.19	612.07	385.77	554.17	785.62	374.77	469.59	60.51	931.09	**0.0007762**	**0.001099**
***PYHIN1***	2.96	2.55	4.18	2.25	2.12	7.40	2.59	2.89	4.10	3.27	3.72	3.76	8.26	2.48	6.53	1.87	3.30	0.22	1.06	3.12	1.96	2.79	0.00	0.00	1.63	1.76	0.93	2.41	**0.0007801**	**0.008206**
***SLA***	17.43	0.54	15.34	17.03	15.80	1.30	11.68	17.86	19.16	12.44	1.19	14.58	20.30	12.12	18.01	12.64	1.55	0.98	0.00	1.51	11.92	1.43	18.16	0.00	0.99	0.00	0.12	0.36	**0.0008736**	**0.170331**
***CD63***	5.13	4.22	4.27	3.53	6.71	6.23	4.04	3.33	0.00	5.66	0.00	3.55	6.38	4.67	4.76	0.00	5.16	0.00	0.00	0.00	3.95	3.61	0.00	3.70	0.00	0.00	0.65	0.85	**0.0009408**	**0.129691**
***SHISAL2A***	1.04	0.71	1.41	1.75	2.23	1.67	0.84	0.94	2.31	1.44	0.89	1.59	1.44	0.65	1.37	0.61	1.23	0.40	0.20	0.46	0.78	1.65	0.00	0.00	0.51	1.57	0.11	0.16	**0.0009501**	**0.000681**
***PDCD11***	1.39	18.32	1.36	1.64	2.37	2.50	15.93	2.39	1.33	1.98	20.23	1.24	3.14	2.08	1.36	12.04	3.00	15.49	15.76	17.59	1.71	25.84	27.87	31.03	16.29	19.35	6.03	19.79	**0.000976**	**0.547628**
***PPP1R16B***	2.04	2.34	1.86	1.31	3.84	3.92	1.73	2.47	2.79	2.84	1.76	2.36	3.90	1.82	2.56	1.02	2.73	0.79	1.04	1.56	1.75	1.78	0.00	1.70	2.21	2.06	0.24	0.82	**0.0009954**	**0.001094**

## Discussion

Despite the poor overall prognosis of pancreatic cancer, patients with high numbers of infiltrating T cells as determined by quantitative immunohistology have improved outcome. To understand the complexity of the tumor immune environment, we performed RNA-Seq and evaluated gene expression-based analyses of tumor-infiltrating cells. We found that there were limitations in current gene expression analyses of infiltrating immune cells, particularly where overall infiltration was low. Both CIBERSORT and xCELL showed poor concordance with IHC, and individual immune cell types identified by gene expression analysis had limited prognostic value. This could be somewhat overcome by aggregating molecularly-identified immune populations, for example into total T cell infiltrates, which showed some correlation to quantitative immunohistology and could be predictive of outcome. To determine if we could identify alternative molecular signatures of highly infiltrated tumors, we performed class comparison of gene expression and identified a transcriptional pattern in pancreatic adenocarcinoma that had predictive value for disease-free survival in the TCGA cohort.

Immunohistology with validated antibodies is the gold standard for quantitative assessment of infiltrating immune cells in cancer [27]. However, the diversity of cell types and limited number of cell-type specific markers makes it difficult to accurately assess many infiltrating cell types using histology. Flow cytometry is more able to address this diversity, using a series of gates to distinguish cell subtypes expressing multiple overlapping markers; however, this cannot be performed with archived tissues. The recent improvement in image analysis and technological improvements in multiplex staining has permitted much more complex assessment of tumors using immunohistology [28]. This is critical since many of our single markers have limitations. For example, our study used CD68 as a well-validated marker for tumor associated macrophages. However, there are significant limitations in the use of CD68 as a sole marker of macrophages in tumors [20], particularly in view of the diverging phenotypes macrophages can generate. In particular, while there may be a spectrum of macrophage phenotypes [29], the polarized M1 (classical) versus M2 (alternative) macrophage phenotype has proven useful in discriminating macrophages that support versus suppress adaptive immunity to tumors [30–33]. Therefore, the presence of macrophages does not necessarily indicate that they generate immune suppression in the tumor.

The recent development of algorithms that can analyze gene expression data to estimate the prevalence of the broad range of cell types in a mixed tissue samples, combined with the increasing affordability of comprehensive genomic profiling of patient tumors, has opened new avenues of research [21]. While gene expression data can provide a great deal of information from small quantities of tissue, there are potential issues in estimating immune cell numbers in pancreatic tumors, since the abundance of some of these populations can be very low even when they have prognostic significance. For example, xCELL gene signatures were identified using purified populations and validated on peripheral blood samples [5], which have a very different immune cell abundance when compared to tumors. CIBERSORT was shown to have superior performance to other approaches available at the time to assess immune infiltrating cells from genomic data [6]; however, performance was limited when the target immune populations represented fewer than 1% of the spiked mixture. Each approach provides valuable information on immune infiltration and may be sufficient to assess the environment of more abundantly infiltrated tumors. However, there are limitations based on the number of RNA reads provided by each infiltrating cell in a bulk population. Immunohistology has its own limitations, particularly those relating to epitope preservation through tissue processing, and the difficulties in standardizing staining over time and between institutions. In this study we set quantitative immunohistology as the gold standard for comparison; however, standard sampling issues such as selection of an appropriate archived tissue block and the relevance of a single 5μM section to the tissue as a whole can lead to inaccuracies that apply to each approach. Novel technologies are emerging that incorporate the geographic information of histology with comprehensive gene profiling, and have the potential to change how we assess the immune complexity of tumors [34]. Further analysis of patients giving discordant RNA and IHC data would be valuable to understand the impact of sampling versus other complicating factors that could explain the variations.

The strength of genomic and other omic profiling is the wealth of data that can be extracted simultaneously. Along with infiltrating cells, omic analyses can inform as to the mutational status of the tumor [8] to identify immunotherapeutic targets [35], and identify expression of inflammatory and chemokine markers that may dictate immune cell recruitment [36, 37]. Such analyses would be best combined with genomic analysis approaches that subdivide patients according to novel molecular subtypes, some of which include tumor subtypes associated with higher immune infiltrates [38, 39]. However, further subdivision of patients will require much larger cohorts to generate meaningful results. All of this can be performed on very small quantities of patient material, at decreasing cost and with increasing speed. While IHC-based multiplexing continues to increase the number of analytes that can be assessed on a single tissue sample, this approach depends on the availability of high-quality validated antibodies for each target. Unbiased sequencing-based approaches are to some degree future-proofed against genes that may be of interest yet do not currently have reagents for IHC or other analyses.

Further refinement of gene expression profiling for pancreatic adenocarcinoma and similar poorly infiltrated tumors could have significant benefits in personalizing immunotherapy for these recalcitrant tumors. For example, we show that CTLA4 is enriched in patients with high T cell infiltration and is part of the gene set that is associated with improved disease free survival. Antibodies targeting CTLA4 are an effective immunotherapy in some tumors [40], but single agent anti-CTLA4 is not effective in patients with locally advanced and metastatic pancreatic adenocarcinoma [41]. In preclinical models of pancreatic adenocarcinoma, we similarly found that anti-CTLA4 is ineffective as a single agent [42]. The combination of anti-CTLA4 and radiation therapy is curative, but only where the host has good pre-existing immunity to the tumor. Thus, gene expression profiling may help identify patient subsets with adequate T cell infiltration that may benefit from immunotherapy combinations, and direct other patients to novel interventions to improve their tumor immune environment prior to further treatment.

## Conclusions

While immunotherapy options are currently limited for patients with pancreatic adenocarcinoma, these and other data showing an impact of immune infiltrates on patient outcomes suggests we should continue to refine our understanding of the immune environment and pursue immune therapies that are appropriate to the particular tumor environments of pancreatic cancer. At present RNASeq-based analyses must take into account the poor overall infiltrate in some tumor types to provide accurate assessments of the tumor environment.

## Supporting information

S1 Fig Pearson correlation matrix of infiltrating immune cells calculated from RNASeq data using CIBERSORT. Rows are centered; no scaling is applied to rows. Both rows and columns are clustered using correlation distance and average linkage.
(PDF)

S2 Fig Pearson correlation matrix of infiltrating immune cells calculated from RNASeq data using xCELL. Rows are centered; no scaling is applied to rows. Both rows and columns are clustered using Manhattan distance and average linkage.
(PDF)

S3 Fig Heatmap showing percent match between genes used in xCell gene signature for each defined cell type. Rows are centered; no scaling is applied to rows. Both rows and columns are clustered using correlation distance and average linkage.
(PDF)

S4 Fig Comparison of all RNA-based approaches to assess infiltrates. a) The immune infiltrate score determined by MCPcounter of i) MCPcounter T cell *vs*. IHC CD3^+^, ii) MCPcounter CD8 T cell *vs* IHC CD8^+^, and iii) MCPcounter monocytic lineage *vs* IHC CD68^+^ cell populations from the same patient. Each symbol represents one patient. b) analysis as in a) for i) EPIC Bref CD8 T cells and ii) EPIC Tref macrophages. c) Pearson correlation matrix of infiltrating T cell subsets calculated from RNASeq data using xCELL, CIBERSORT, MCPcounter, EPIC Bref, and EPIC Tref. Rows are centered; no scaling is applied to rows. Both rows and columns are clustered using Manhattan distance and average linkage.
(PDF)

S1 Table Gene-based expression levels for all patients with RNASeq analysis in the study, along with quantitative IHC. (TXT)
