# Supplementary material for: Transcriptional and immunohistological assessment of immune infiltration in pancreatic cancer
Source: PLoS One. 2020 Aug 31;15(8):e0238380. doi: 10.1371/journal.pone.0238380 (PMC7458344; doi:10.1371/journal.pone.0238380)
Supplement: S1 Fig — Rows are centered; no scaling is applied to rows. Both rows and columns are clustered using correlation distance and average linkage. (PDF) [file pone.0238380.s001.pdf]

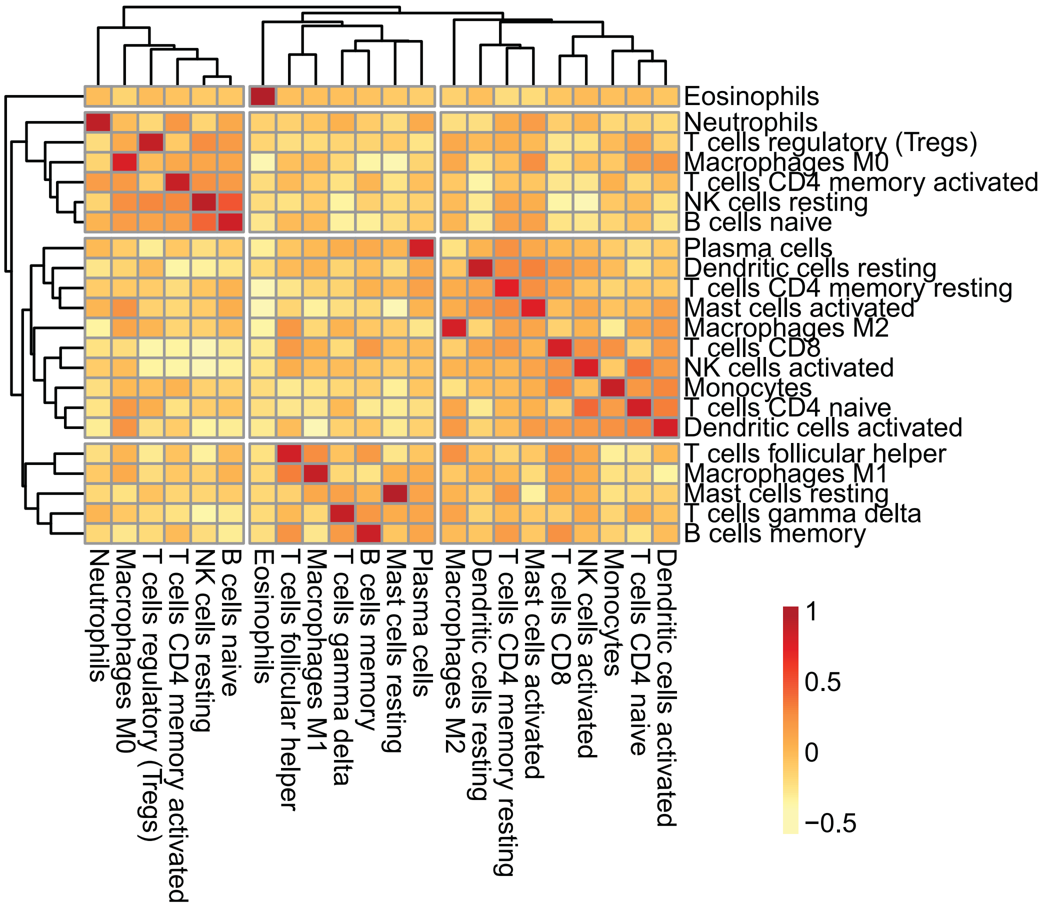

Supplementary Figure 1. Pearson correlation matrix of infiltrating immune cells calculated from RNASeq data using CIBERSORT.

Rows are centered; no scaling is applied to rows. Both rows and columns are clustered using correlation distance and average linkage. 22 rows, 22 columns.
