# Supplementary material for: Transcriptional and immunohistological assessment of immune infiltration in pancreatic cancer
Source: PLoS One. 2020 Aug 31;15(8):e0238380. doi: 10.1371/journal.pone.0238380 (PMC7458344; doi:10.1371/journal.pone.0238380)
Supplement: S3 Fig — Rows are centered; no scaling is applied to rows. Both rows and columns are clustered using correlation distance and average linkage. (PDF) [file pone.0238380.s003.pdf]

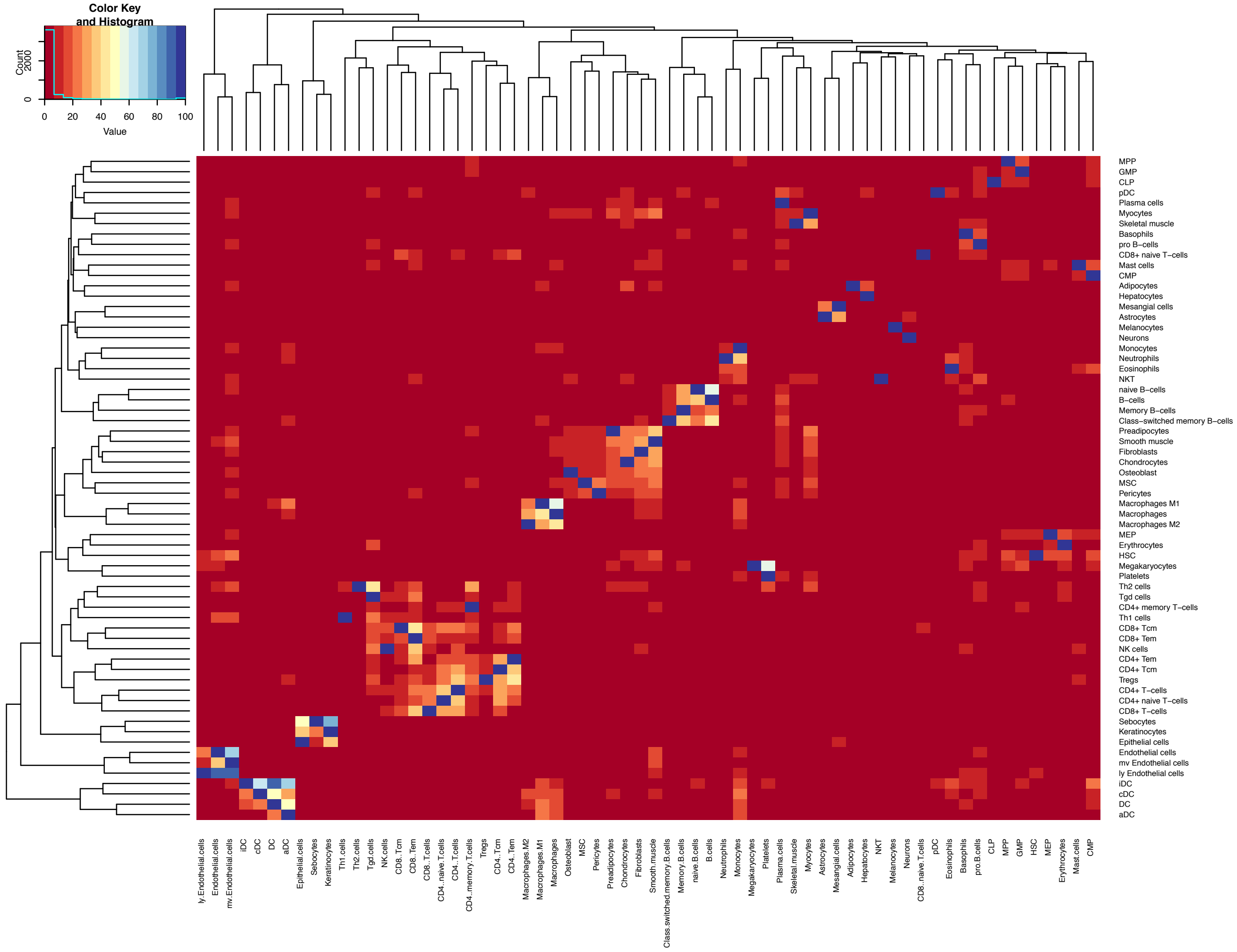

Supplementary Figure 2. Heatmap showing percent match between genes used in xCell gene signature for each defined cell type.
