# Supplementary material for: Transcriptional and immunohistological assessment of immune infiltration in pancreatic cancer
Source: PLoS One. 2020 Aug 31;15(8):e0238380. doi: 10.1371/journal.pone.0238380 (PMC7458344; doi:10.1371/journal.pone.0238380)
Supplement: S4 Fig — a) The immune infiltrate score determined by MCPcounter of i) MCPcounter T cell vs. IHC CD3+, ii) MCPcounter CD8 T cell vs IHC CD8+, and iii) MCPcounter monocytic lineage vs IHC CD68+ cell populations from the same patient. Each symbol represents one patient. b) analysis as in a) for i) EPIC Bref CD8 T cells and ii) EPIC Tref macrophages. c) Pearson correlation matrix of infiltrating T cell subsets calculated from RNASeq data using xCELL, CIBERSORT, MCPcounter, EPIC Bref, and EPIC Tref. Rows are centered; no scaling is applied to rows. Both rows and columns are clustered using Manhattan distance and average linkage. (PDF) [file pone.0238380.s004.pdf]

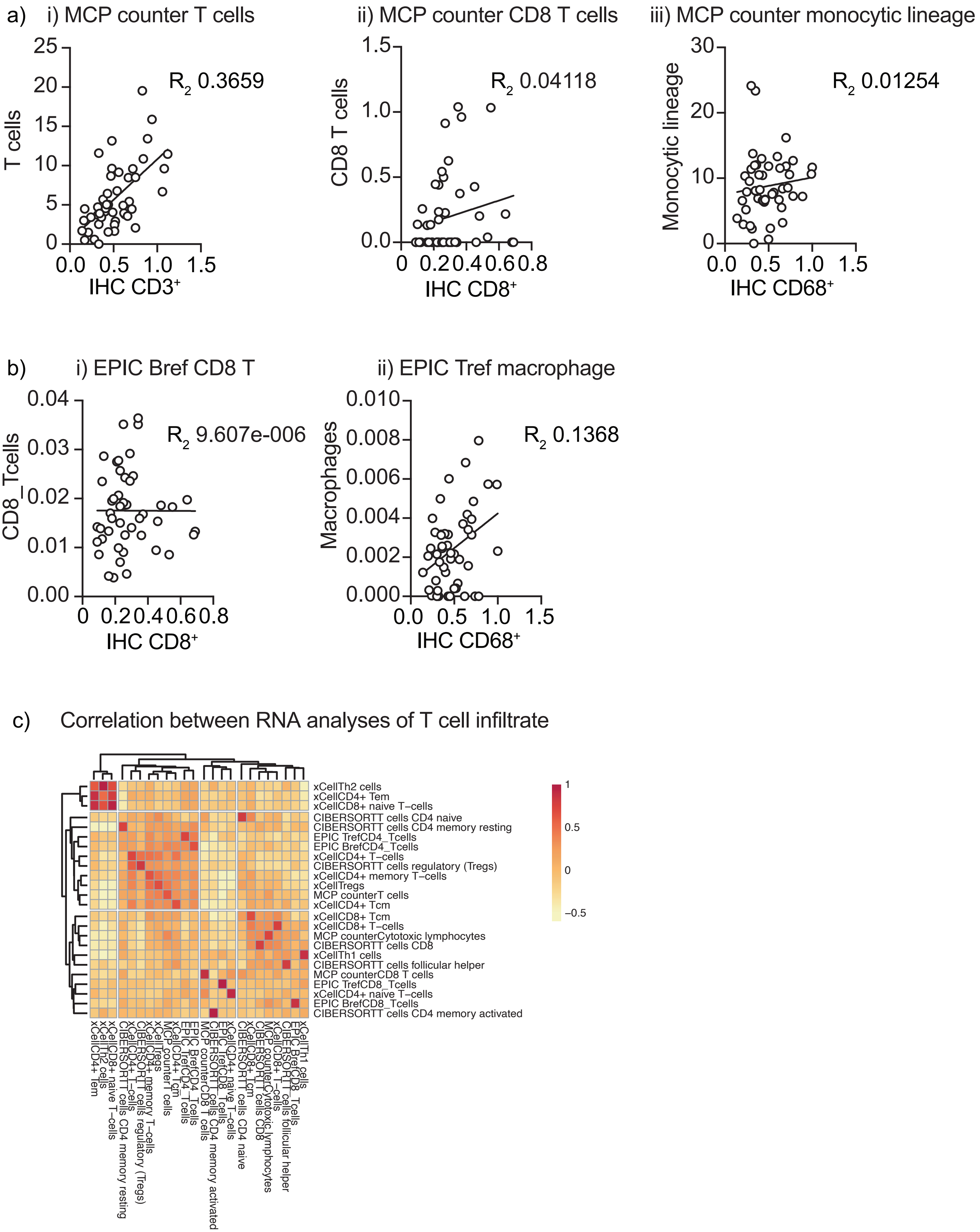
a)
i) MCP counter T cells
T cells
R2 0.3659
IHC CD3+
ii) MCP counter CD8 T cells
CD8 T cells
R2 0.04118
IHC CD8+
iii) MCP counter monocytic lineage
Monocytic lineage
R2 0.01254
IHC CD68+
b)
i) EPIC Bref CD8 T
CD8_Tcells
R2 9.607e-006
IHC CD8+
ii) EPIC Tref macrophage
Macrophages
R2 0.1368
IHC CD68+
c)
Correlation between RNA analyses of T cell infiltrate
xCellTh2 cells
xCellCD4+ Tem
xCellCD8+ naive T-cells
CIBERSORTT cells CD4 naive
CIBERSORTT cells CD4 memory resting
EPIC TrefCD4_Tcells
EPIC BrefCD4_Tcells
xCellCD4+ T-cells
CIBERSORTT cells regulatory (Tregs)
xCellCD4+ memory T-cells
xCellTregs
MCP counterT cells
xCellCD4+ Tcm
xCellCD8+ Tcm
xCellCD8+ T-cells
MCP counterCytotoxic lymphocytes
CIBERSORTT cells CD8
xCellTh1 cells
CIBERSORTT cells follicular helper
MCP counterCD8 T cells
EPIC TrefCD8_Tcells
xCellCD4+ naive T-cells
EPIC BrefCD8_Tcells
CIBERSORTT cells CD4 memory activated
1
0.5
0
-0.5
